# Definition and characterization of novel HLA-*A02-restricted CD8^+^ T cell epitopes derived from JCV polyomavirus with clinical relevance

**DOI:** 10.18632/oncotarget.12387

**Published:** 2016-10-01

**Authors:** Jiju Mani, Lei Wang, Angela G. Hückelhoven, Anita Schmitt, Alma Gedvilaite, Nan Jin, Christian Kleist, Anthony D Ho, Michael Schmitt

**Affiliations:** ^1^ Department of Internal Medicine V, Heidelberg University Hospital, Heidelberg, Germany; ^2^ Department of Eukaryote Genetic Engineering, Institute of Biotechnology, Vilnius University, Vilnius, Lithuania; ^3^ Department of Hematology, ZhongDa Hospital, Southeast University, Nanjing, P. R. China; ^4^ Department of Transplantation Immunology, Heidelberg University Hospital, Heidelberg, Germany; ^5^ Department of Nuclear Medicine, Heidelberg University Hospital, Heidelberg, Germany

**Keywords:** JCV, T cell epitopes, progressive multifocal leukoencephalopathy, virus like particles, immunotherapy

## Abstract

Human JC and BK polyomaviruses (JCV/BKV) can establish a latent infection without any clinical symptoms in healthy individuals. In immunocompromised hosts infection or reactivation of JCV and BKV can cause lethal progressive multifocal leukoencephalopathy (PML) and hemorrhagic cystitis, respectively. Vaccination with JCV/BKV derived antigen epitope peptides or adoptive transfer of virus-specific T cells would constitute an elegant approach to clear virus-infected cells. Furthermore, donor leukocyte infusion (DLI) is another therapeutic approach which could be helpful for patients with JCV/BKV infections.

So far, only few immunodominant T cell epitopes of JCV and BKV have been described and therefore is a fervent need for the definition of novel epitopes. In this study, we identified novel T cell epitopes by screening libraries of overlapping peptides derived from the major capsid protein VP1 of JCV. Virus like particles (VLPs) were used to confirm naturally processing. Two human leucocyte antigen (HLA)-A*02-restricted epitopes were characterized by fine mapping with overlapping peptides and nonamer peptide sequences were identified. Cytokine release profile of the epitope-specific T cells was analyzed by enzyme-linked immunospot (ELISPOT) assays and by flow cytometry. We demonstrated that T cell responses were of polyfunctional nature with the potential of epitope-specific killing and cross-reactivity between JCV and BKV. These novel epitopes might constitute a new potential tool to design effective diagnostic and therapeutic approaches against both polyomaviruses.

## INTRODUCTION

JC and BK polyomaviruses (JCV/BKV) are double stranded DNA viruses which can reactivate in the immunocompromised host and cause severe if not even lethal disease [1, 2].

Reactivation of JCV may result in a fatal central nervous system disease called progressive multifocal leukoencephalopathy (PML). PML commonly occurs in patients with HIV infection (80%), and less frequently in patients with hematologic malignancies (13%) or organ transplant patients (5%) [3–6]. BKV is the causative agent of hemorrhagic cystitis and shares 75% identity of the genome with JCV. The major capsid protein VP1 is considered to be among the most immunogenic proteins of polyomaviruses [7]. The sequences of VP1 proteins derived from JCV/BKV are 78% identical.

Two immunodominant human leukocyte antigens (HLA)-A*0201-restricted epitopes derived from VP1-protein have been characterized in PML patients (JCV-VP1-p36-44 SITEVECFL and JCV-VP1-p100-108 ILMWEAVTL). Interestingly, cross-reactivity of T cells towards homologous epitopes of BKV VP1 (BKV-VP1-p44-52 AITEVECFL and BKV-VP1-p108-116 LLMWEAVTV) was described [7–9]. The cross-reactivity was demonstrated in-terms of cross-killing experiments and identification of epitopes derived from both viruses by corresponding multimers [7–10]. Therefore it is highly likely that a successful T cell therapy against JCV infection is also effective against BKV infection.

However, due to the inadequate availability of effective anti-viral drugs, the treatment of PML is largely dependent on the restoration of the immune system of the host. Adoptive T cell transfer is one method which has been practiced since 1990s for effective reconstitution of the immune system. Adoptive immunotherapy with Epstein-Barr virus- (EBV) [11, 12], cytomegalovirus- (CMV) [13–15], adenovirus- [16, 17] and JCV-specific [18] peripheral blood mononuclear cells (PBMCs)/T cells have shown successful clinic results. During antigen-specific T cell therapy, presence of allo-reactive T cells can have detrimental effects in patients due to graft-versus-host disease. In this context, it is now possible to select pure virus-specific T cells by their ability to secrete cytokines [19–21], by major histocompatibility complex (MHC)-multimers [22, 23] and recombinant T cell receptor technology. However, in the case of JCV, the repertoire of immunodominant antigens or T cell epitopes is very limited. Therefore, there is a fervent need to enrich this armamentarium with further T cell epitopes derived from BKV/JCV. This could also enrich the options to use virus-specific donor leukocyte infusion (DLI) for patients with JCV/BKV reactivation.

In this study, we aimed at mapping the CD8^+^ T cell epitopes by using overlapping pentadecamer peptides derived from the VP1-protein. Furthermore, we used virus like particles (VLPs) derived from VP1-protein of JCV. Due to structural and immunological similarities with the natural virus, VLPs served as an important tool for the confirmation of natural processing of identified T cell specificities. We have identified several novel T cell specificities, out of which two HLA-A*02 T cell epitopes were characterized in healthy donors.

## RESULTS

### JCV VP1-specific CD8^+^ T cell responses in healthy donors

To measure JCV-specific T cell responses towards the VP1-protein of JCV, IFN-γ ELISPOT assays were performed using a total of 86 VP1-spanning overlapping pentadecamer peptides (OP). In order to expand CD8^+^ T cells, mixed lymphocyte peptide culture (MLPC) assays were performed with magnetically sorted CD8^+^ T cells as responders and irradiated CD8^-^ PBMCs as stimulators. Antigen-specific responses were characterized by the stimulation index (S.I). and responses were considered positive if S.I. was equal or more than two.

**Figure 1:**
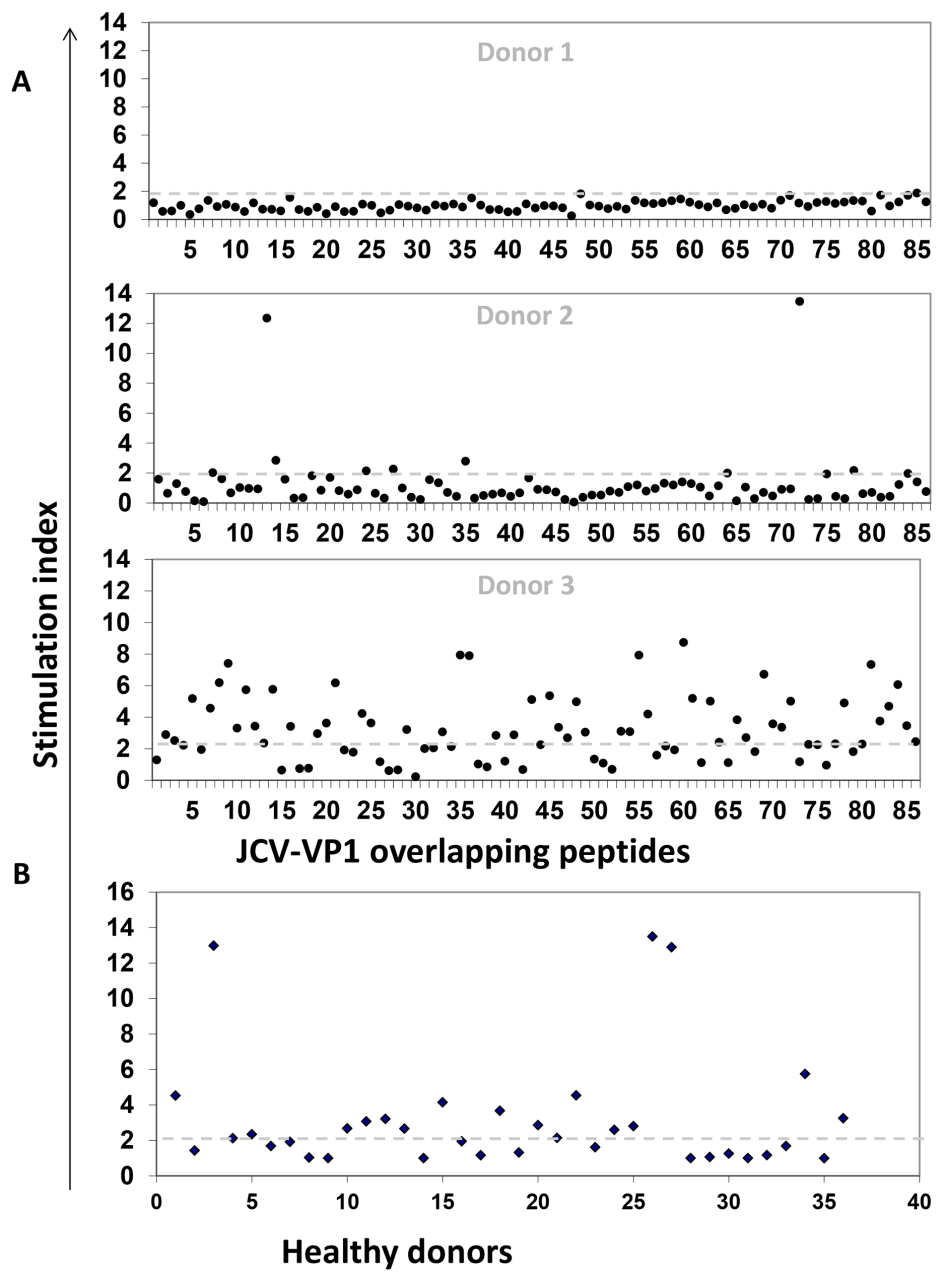
JCV-specific T cell responses of healthy donors In order to expand CD8^+^ T cells, MLPC assays were performed with magnetically sorted CD8^+^ T cells as responder and irradiated CD8^-^ PBMCs or T2 cells as stimulators. Antigen-specific responses were characterized by a stimulation index (S.I.). S. I. representing the ratio of the number of IFN-γ secreting cells after antigen stimulation to that without stimulation (negative control) in ELISPOT assays. Responses were considered as positive when the S.I. was equal or above the cut-off of two (dashed grey line). **A**. Dot-plots showing the breadth of JCV-specific T cell responses represented by three healthy donors (Donor 1, 2 and 3). Cells were stimulated with 86 individual overlapping peptides derived from VP1 protein of JCV. **B**. Dot-plot showing production of IFN-γ in 36 HLA-A*02 positive healthy donors after stimulating with VP1 peptide pool (overlapping 1 to 86 peptides in one pool). 16 donors out of 36 exhibit positive T cell responses.

We observed variable CD8^+^ T cell responses in the peripheral blood from healthy donors when cells were stimulated with individual overlapping peptides. Data of three different donors are illustrated in Figure 1. While some donors did not respond to any peptide (Figure 1A, donor 1), others showed IFN-γ release in response to most of the individual peptides (Figure 1A, donor 3). Few healthy donors even generated strong responses to selected peptides (Figure 1A, donor 2).

Moreover, we also measured frequencies of VP1-protein-specific responses by using a VP1-peptide pool in 36 HLA-A*02-positive donors after MLPC in IFN-γ ELISPOT (Figure 1B). T2 cells, expressing the allele HLA-A*02 on their cell surface, were used as stimulators, therefore only HLA-A*02-specific T cell responses could be observed. Around 44% of the healthy donors were tested positive. The results demonstrate the range of JCV-specific T cell responses.

### Identification of naturally processed overlapping pentadecamer peptides

**Figure 2:**
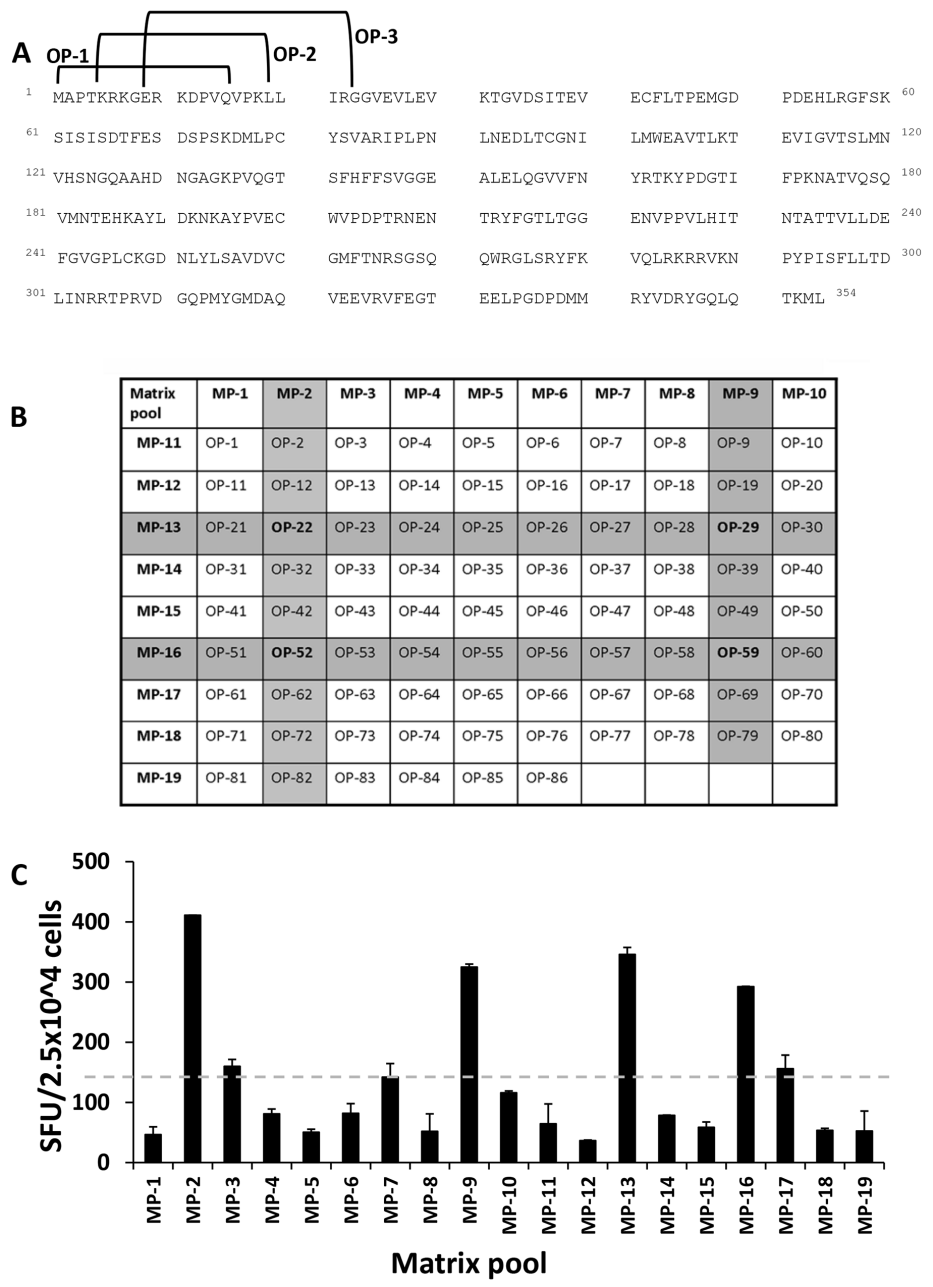
Strategy for the identification of naturally processed pentadecamer peptides In order to identify naturally processed peptides, VP1-derived VLPs were used as a primary stimulus followed by VP1-derived overlapping peptide stimulation in MLPC assays with PBMCs from healthy donors. In the experimental setting, T2 cells served as APCs in ELISPOT assays which confirmed the HLA-A*02-restriction of identified potential peptide epitopes. **A**. The JCV VP1-protein (GenBank Accession No. AFH57194.1) [62] consists of 354 amino acids and was covered by 86 peptides shifted about four amino acids so that the peptides had a consecutive overlap of eleven amino acids. Exemplarily the peptides OP-1 to OP-3 are marked. OP-1 consists of amino acid 1 to 15, OP-2 of amino acid 5 to 19 and OP-3 of amino acid 9 to 23. **B**. These overlapping peptides were placed in a matrix grid consisting of 19 matrix pools (MP) in such a way that each peptide is uniquely contained in two intersecting pools. **C**. One representative example for one HLA-A*02-positive donor out of 12 healthy donors is shown. Positivity in the assay was defined as 2-fold beyond background (neg. control = 155 spots). The dashed grey line marked the background. Cells were evaluated in triplicates in each ELISPOT assay and mean and standard deviations were calculated. In this example, four MP (MP-2, MP-9, MP-13 and MP-16) were positive which corresponded to four overlapping peptides OP-22, OP-29, OP-52 and OP-59. SEB served as a positive control resulting in approximately 2,000 SFU/5 × 10^4^ CD8^+^ cells.

**Figure 3:**
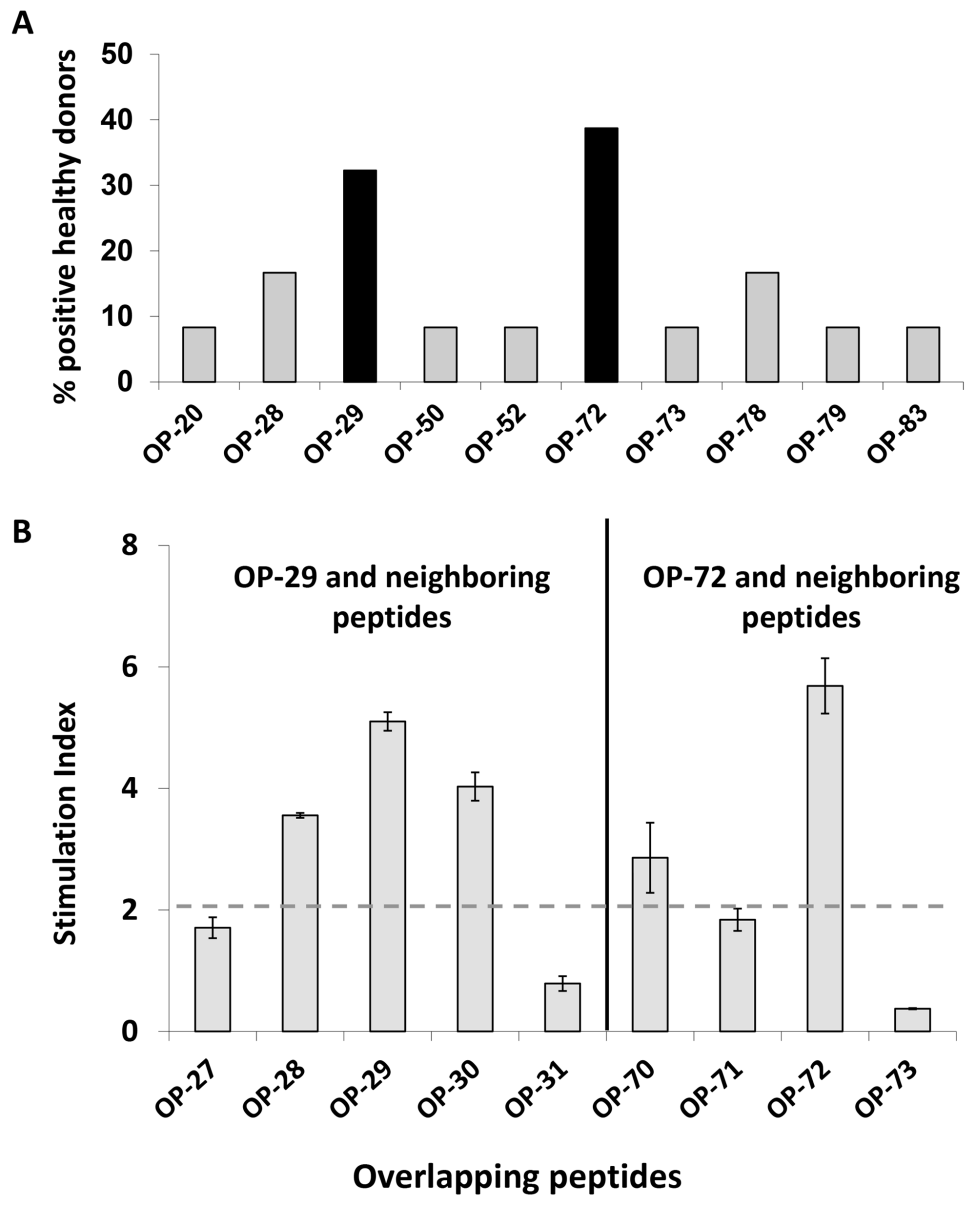
Identification of immunodominant pentadecamer peptides To identify immunodominant T cell epitopes, 31 HLA-A*02^+^ healthy donors were stimulated with selected peptides. **A**. Two pentadecamer peptides (black bars) were found to be positive in more than 30% of all tested donors. **B**. IFN-γ production by CD8^+^ T cells in response to individual neighboring overlapping peptides confirmed the presence of immunogenic sequences within OP-29 and OP-72. Triplicates for each peptide were analyzed in ELISPOT assays. Mean and standard derivations were calculated. Antigen-specific responses were characterized by a stimulation index (S.I.). S. I. representing the ratio of the number of IFN-γ secreting cells after antigen stimulation to that without stimulation (negative control). Responses were considered as positive when the S.I. was equal or above the cut-off of two (dashed grey line).

In order to identify naturally processed peptides, JCV VP1-derived VLPs were used as a primary stimulus followed by VP1 overlapping peptide stimulation in MLPC. Additionally, T2 cells served as antigen presenting cells (APCs) in ELISPOT assays which confirmed the HLA-A*02 restriction of identified potential peptides. A matrix format with the 86 overlapping peptides in a horizontal and vertical grid was used to further reduce the number of cells in each experimental setting. To this end, 19 matrix pools (MP) were generated with each individual peptide contained in two different MP (Figure 2A and 2B). VP1-protein derived from JCV produced in self-assemble into stable VLPs [24]. In this study, after MLPC with VLPs and subsequent stimulation using MP in the ELISPOT assays, solely naturally processed T cell specificities were identified. As seen in a representative example (Figure 2C), for one HLA-A*02-positive donor, four naturally processed peptides (OP-22, OP-29, OP-52 and OP-59) were identified. By following this protocol, several pentadecamer peptides were identified in 12 tested healthy donors (Table 1). 31 healthy donors were screened with the identified immunogenic pentadecamer peptides. The frequency of responses among the tested healthy donors varied for the different pentadecamer peptides. Immune responses against six of the tested pentadecamer peptides seen in less than 10 % of tested healthy donors whereas immune responses against two other pentadecamer peptides were observed in around 15 % of the tested healthy donors. Responses against the two pentadecamer peptides OP-29 and OP-72 were observed in more than 30% of the 31 tested HLA-A*02-positive donors and thus are considered as immunodominant T cell specificities (Figure 3A). Peptide epitopes which are common represent ideal possible candidates for the development of adoptive T cell therapy approaches for a broader range of patients. Therefore, we focused on these two peptides to further identify the ideal epitope and to characterize the epitope-specific T cells. The newly identified immunodominant T cell epitopes were subsequently confirmed by using neighboring peptides leading to the position of the epitope present within the respective pentadecamer peptide (Figure 3B). Stimulation was performed by using neighboring peptides of OP-29 (OP-27 to OP-31) and OP-72 (OP-70 to OP-73). For example, strong responses to OP-72 but not to the other neighboring peptides (OP-70, OP-71 or OP-73), indicate that the actual epitope is present only in OP-72 (Figure 3B).

**Table 1:** Naturally processed overlapping pentadecamer peptides of JCV-VP1 identified by HLA-A*02 positive healthy donors

Overlapping JCV-VP1 peptide sequences		
Peptide no.	Amino acid position	Peptide sequence*
OP9	33-47	GVDSITEVECFLTPE
OP20	77-91	MLPCYSVARIPLPNL
OP25	97-111	CGNILMWEAVTLKTE
OP28	109-123	KTEVIGVTSLMNVHS
OP29	113-127	IGVTSLMNVHSNGQA
OP50	197-211	PVECWVPDPTRNENT
OP52	205-219	PTRNENTRYFGTLTG
OP72	285-299	KRRVKNPYPISFLLT
OP73	289-303	KNPYPISFLLTDLIN
OP78	309-323	VDGQPMYGMDAQVEE
OP79	313-327	PMYGMDAQVEEVRVF
OP83	329-343	GTEELPGDPDMMRYV

* Known epitope sequences are underlined.

### Fine mapping of identified HLA-A*02 restricted T cell specificities

**Figure 4:**
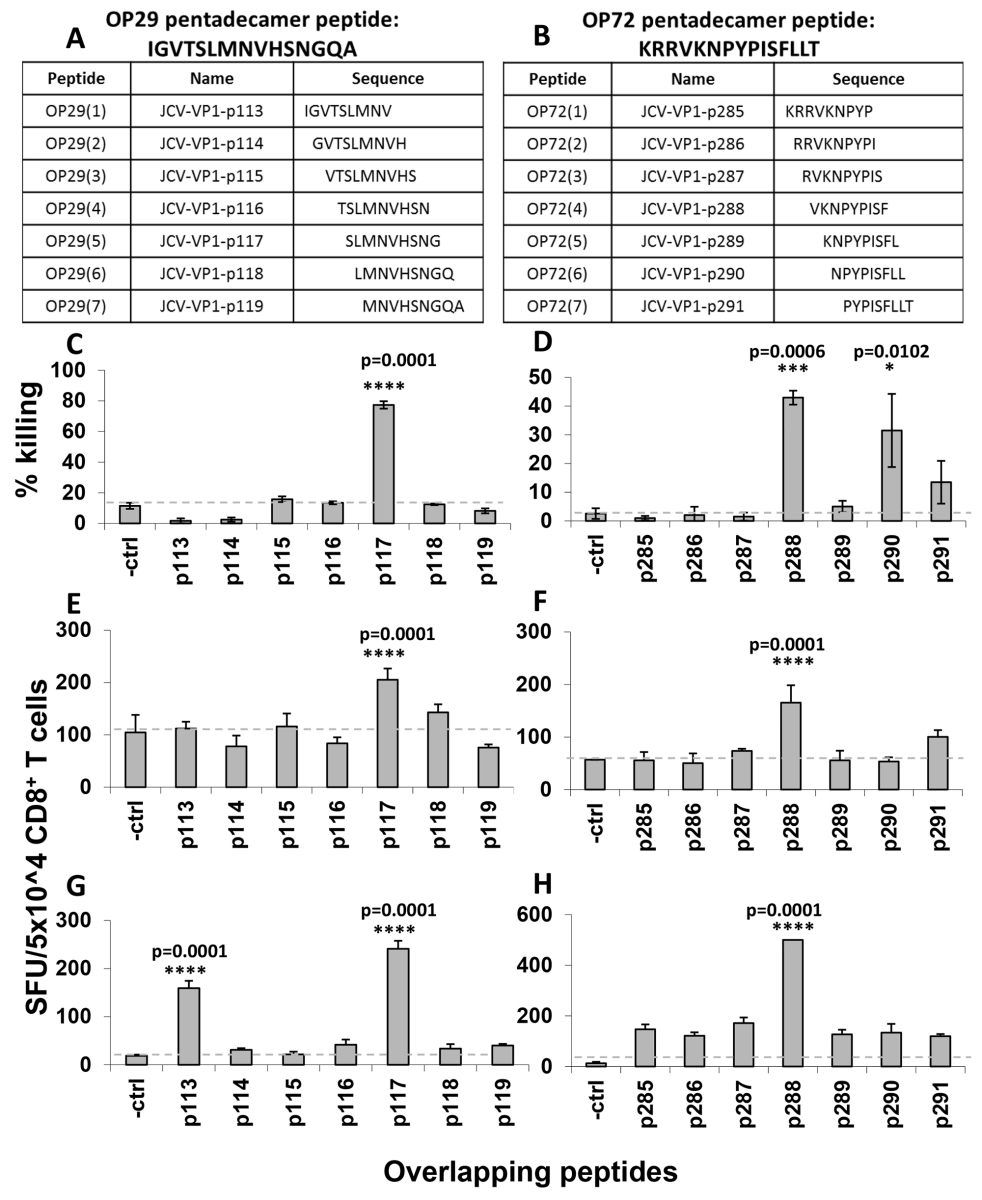
Fine mapping analysis of immunodominant pentadecamer peptides using IFN-γ ELISPOT assays and cytotoxicity assays To verify the HLA-A*02-restricted T cell determinants present within immunodominant peptides, a total of seven nonamer peptides with an eight amino acid overlap were synthesized for both peptides, OP-29 **A**. and OP-72 **B**. and tested individually in cytotoxicity **C, D**. and ELISPOT **E-H**. assays in three healthy donors. T2 cells served as APCs. IFN-γ secretion results are displayed as spot forming units (SFU) per 50,000 CD8^+^ T cells. (A, B) The sequences of overlapping nonamer peptides for OP-29 (A) and OP-72 (B) are displayed. (C, D) The killing potential of CD8^+^ T cells towards T2 cells loaded with a set of nonamer peptides derived from OP-29 (C) and OP-72 (D) are presented. (E-H) ELISPOT assays of two healthy donors with low reactivity (E, F) and high reactivity (G, H) to nonamer peptides derived from OP-29 (JCV-p113 to p119) (E, G) and OP-72 (JCV-p285 to p291) (F, H) are shown. The dashed grey line marked the background. Cells were evaluated in triplicates in each assay and mean and standard deviations were calculated. For statistical analysis we used the one-way ANOVA and compared all conditions with the negative control. A p value <0.05 was considered as significant.

In order to identify HLA-A*02-restricted T cell epitopes, a total of seven nonamer peptides with an overlap of eight amino acids were synthesized from each immunodominant T cell specificity (OP-29 and OP-72) (Figure 4A, 4B). MLPC and ELISPOT assays were performed using T2 cells as APCs with each individual peptide. As it is displayed in Figure 4E-4H for both T cell specificties one immunodominant peptide turned out to be the CD8^+^ T cell determinants that stimulate secretion of IFN-γ. The nonamer peptide of OP-29 is JCV-VP1-p117 (Figure 4C, 4E, 4G) and JCV-VP1-p288 for OP-72 (Figure 4D, 4F, 4H).

To select an epitope as a potent vaccine target, killing activity of T cells towards cellular targets presenting antigenic peptides should also be considered. Therefore, we performed cytotoxicity assays and observed high killing responses after stimulation with the peptides JCV-p117 and JCV-p288, respectively. (Figure 4C, 4D).

In the process of T cell epitope identification, VLP-elicited CD8^+^ T cells were restimulated with newly identified T cell determinants (JCV-p117 and JCV-p288). As the T cells showed functional reactivity, we concluded that both the nonamer peptides JCV-p117 and JCV-p288 were naturally processed and presented by the HLA-A*02 allele.

### CD8^+^ T cells targeting novel, naturally processed determinants secrete different cytokines

We evaluated whether CD8^+^ T cells, sensitized towards novel epitopes, are capable to secrete IFN-γ, TNF-α and IL-2 cytokines upon restimulation. Functional CD8^+^ T cells were differentiated into naïve (T_N_; CD45RA^+^, CCR7^+^), effector memory (T_EM_; CD45RA^-^, CCR7^-^) and effector memory RA^+^/effector (T_E/EMRA_; CD45RA^+^, CCR7^-^) populations based on their expression of the cell surface markers CCR7 and CD45RA [25].

**Figure 5:**
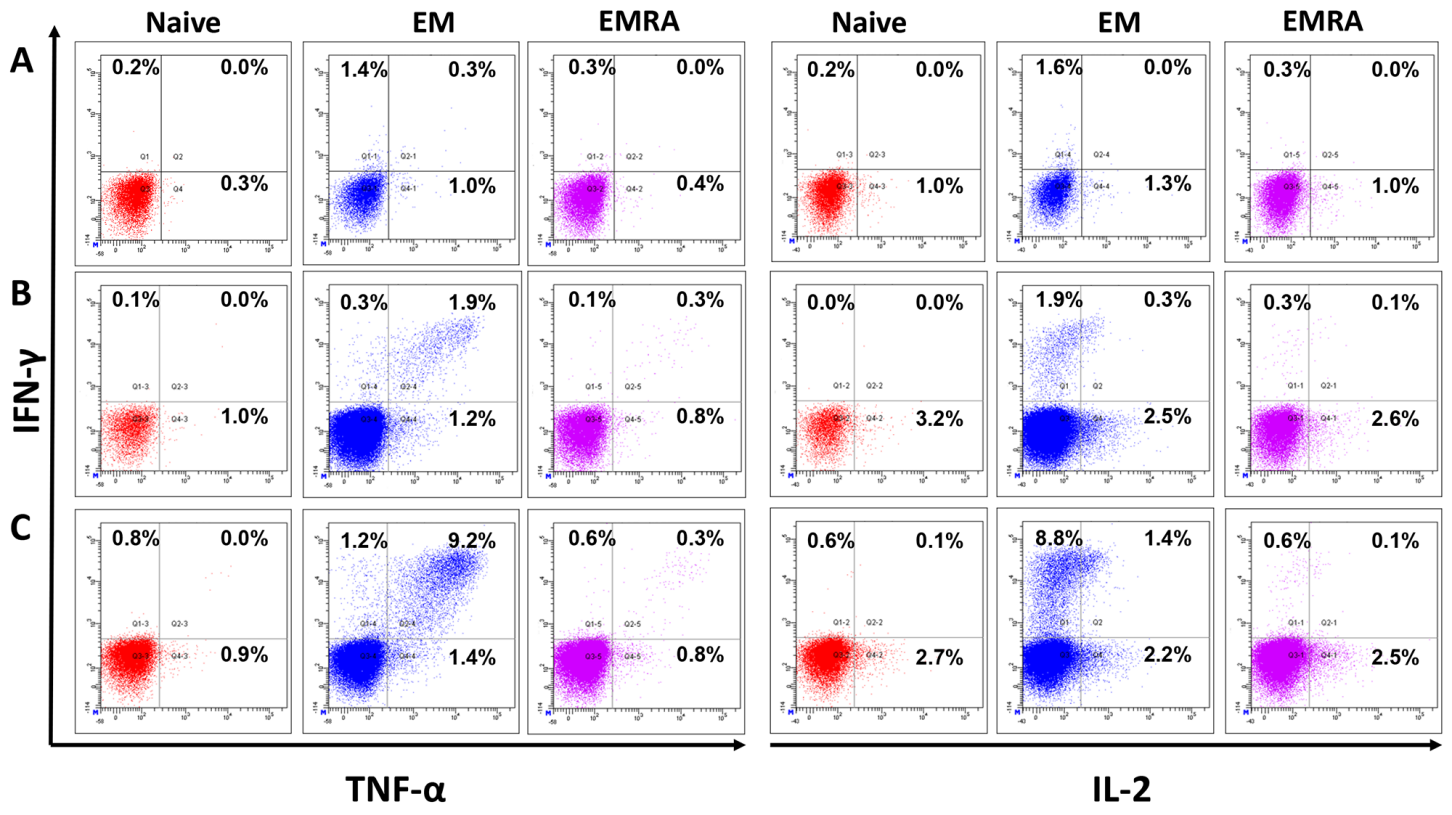
Multifunctional JCV-specific CD8+ T cells: The ability of epitope-specific CD8+ T cells to secrete the cytokines IFN-γ, TNF-α and IL-2 after stimulation with peptides was evaluated by intracellular flow cytometry staining One representative flow cytometry data-set out of four independent experiments is displayed. The functional CD8^+^ T cells were divided into naïve (T_N_), effector memory (T_EM_) and effector memory RA^+^ (T_E/EMRA_) populations based on the expression of cell surface markers, CCR7 and CD45RA, respectively. First, cells were gated on lymphocytes and dead cells were excluded using the live/dead stain, NEAR. Cells were then gated on CD8^+^ T cells and subsequently either on the naïve (CCR7^+^, CD45RA^+^), the effector memory (EM) (CCR7^-^ CD45RA^-^) or the effector memory RA (EMRA) (CCR7^-^ CD45RA^+^). The cells were analyzed for intracellular expression of the inflammatory cytokines IFN-γ, TNF-α and IL-2. CD8^+^ T cells were stimulated and restimulated with T2 cells loaded with: **A**. no peptide, **B**. JCV-p117 nonamer and **C**. JCV-p288 nonamer peptides, respectively. Flow cytometry dot-plots were analyzed for the co-expression of IFN-γ and TNF-α (left panel) and IFN-γ and IL-2 (right panel) in all three T cell subpopulations.

**Figure 6:**
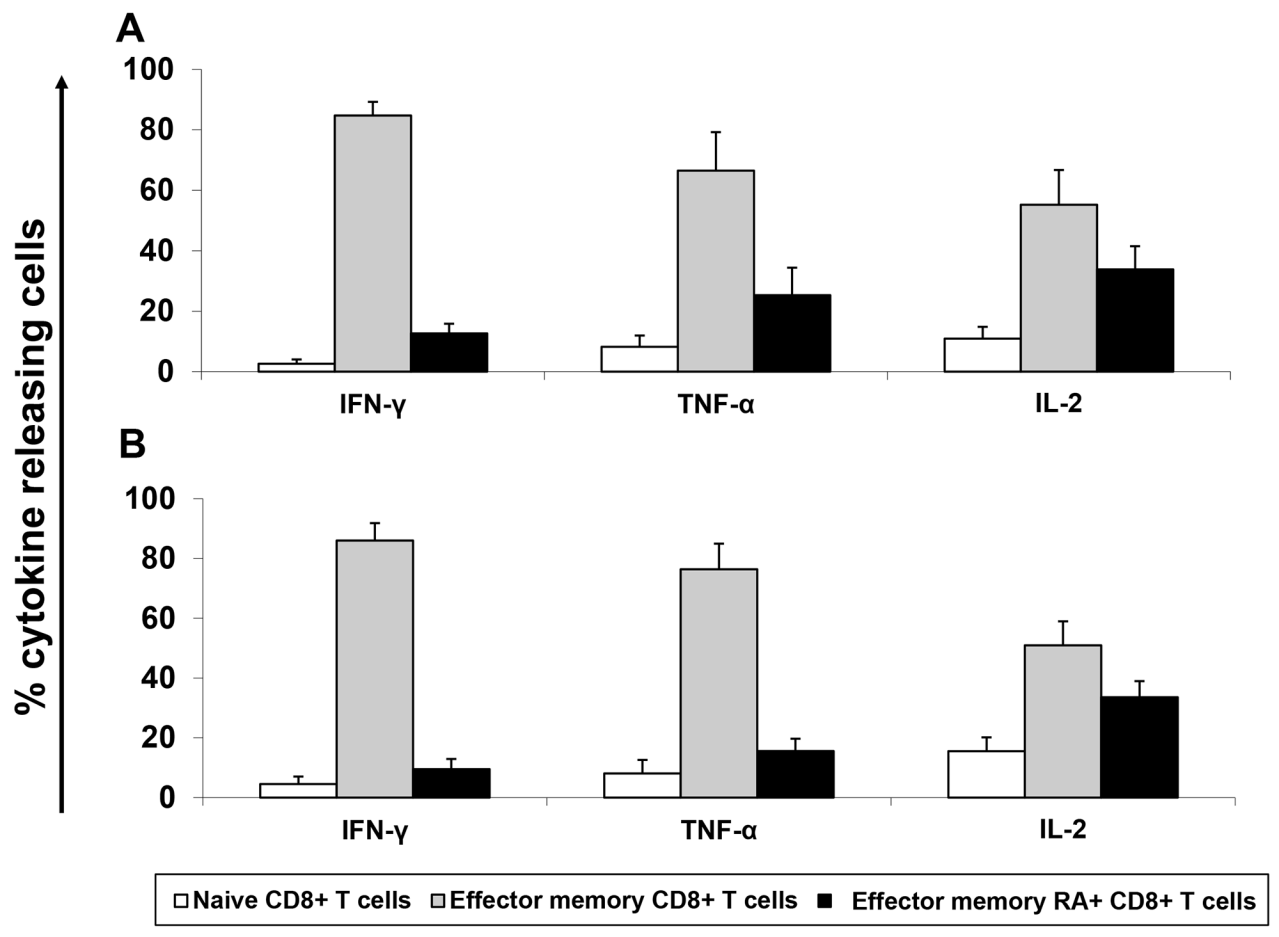
Epitope-specific effector memory CD8+ T cells show a dominant T cell response compared to other cell-population based on their cytokine profile: Bar graphs represent percentage of each subpopulation secreting IFN-γ, TNF-α and IL-2 in positive healthy donors (n=4) Calculation was performed by normalizing the total expression of cytokines for each T cell population. Mean and standard derivations were calculated. Cytokine expression was measured after stimulation with **A**. JCV-p117 nonamer and **B**. JCV-p288 nonamer peptides, respectively.

MLPC assays were performed for one week. Afterwards restimulation was performed for six hours in the presence of Brefeldin A followed by intracellular cytokine staining. Un-stimulated CD8^+^ T cells were used as negative control. IFN-γ, TNF-α and IL-2 secreting CD8^+^ T cells were analyzed in the T cell subpopulations by flow cytometry in four different donors (Figure 5). When compared to the negative control, CD8^+^ T cells stimulated with peptides JCV-p117 and JCV-p288 showed higher expression of all three cytokines (Figure 5). In all tested donors, predominantly the effector memory T cell (T_EM_) subpopulation, CD45RA^-^ (antigen sensitized T cells) and CCR7^-^ (memory population), secreted type-1 cytokines (Figure 5). Figure 6 showed the relative percentages for each subpopulation of CD8^+^ T cells and for each cytokine. The majority of the IFN-γ and TNF-α secreting population was double positive (Figure 5), what is a characteristic of polyfunctional T cells. Polyfunctional T cell responses are considered to be crucial in effective infection control and positive outcomes in clinical settings.

### CD8^+^ T cells targeting novel JCV epitopes are cross-reactive against JCV and BKV species

**Figure 7:**
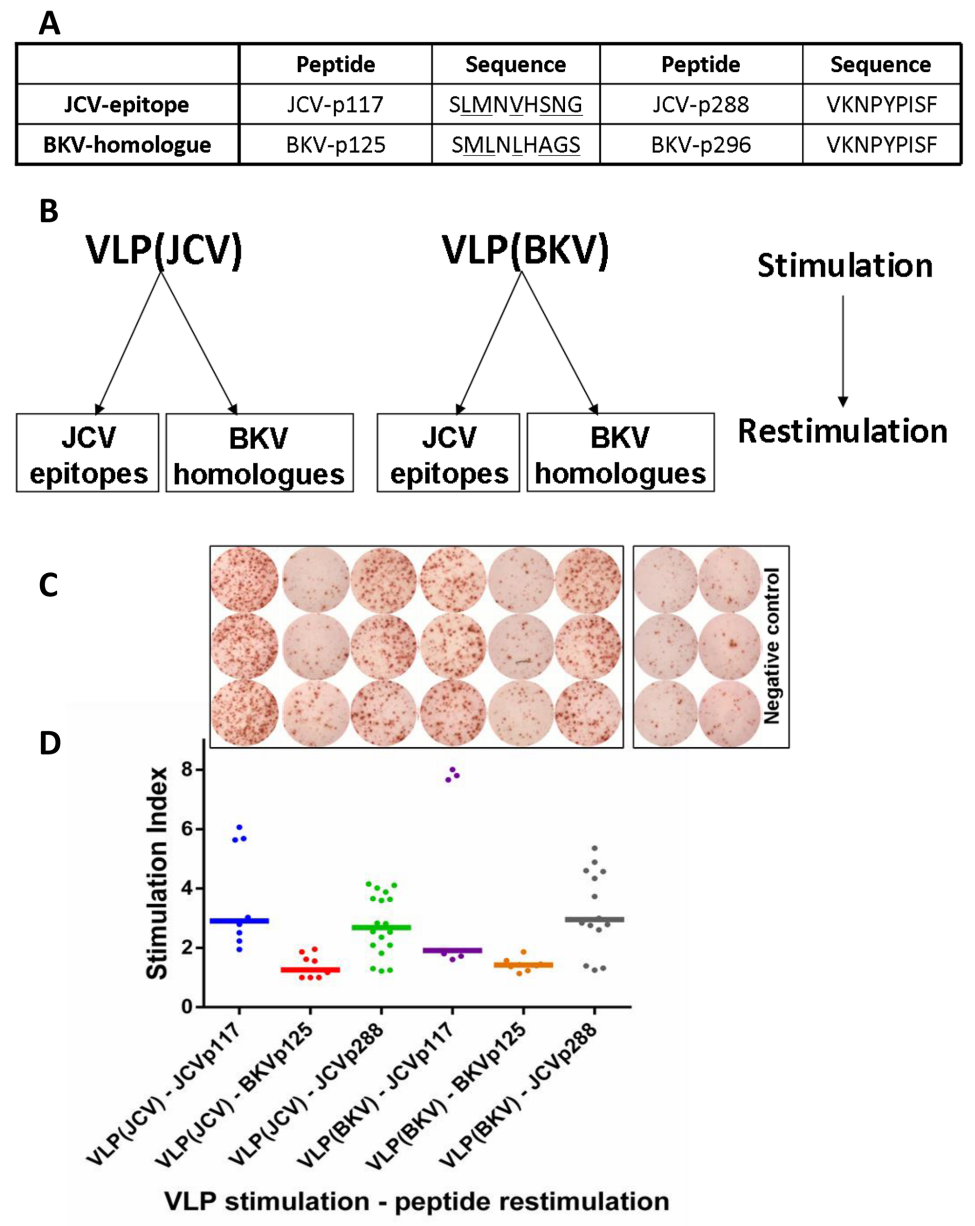
JCV-specific CD8+ T cells are cross-reactive towards VP1 protein VLPs derived from both JCV and BKV **A**. BKV homologous sequences corresponding to JCV-epitopes p117 and p288 are displayed. Amino acid mismatches between JCV and BKV peptides are underlined. **B**. To assess cross-reactivity of CD8^+^ T cells, criss-cross experiments were performed using VP1 VLPs as first stimulus and peptides as second stimulus. **C**. IFN-γ ELISPOT data shows activated p117 and p288-specific T cells after stimulation with both JCV and BKV VP1 VLPs representative for one donor. Cells were evaluated in triplicates in each ELISPOT assay and mean was calculated. **D**. The corresponding dot-plot represents IFN-γ secretion expressed by stimulation index and horizontal bars displaying the median value for each set. The abbreviations on the x-axis define 1^st^ and 2^nd^ stimuli. For example “VLP(JCV)-JCVp117 means VLP(JCV) as 1st and JCVp117 peptide as 2nd stimulus. Altogether six healthy donors were tested.

To confirm cross-reactivity against JCV and BKV, BKV sequences homologous to the JCV epitopes p117 and p288 were synthesized. While JCV-p288 and the corresponding BKV sequence (BKV-p296) were identical, JCV-p117 and BKV sequence (BKV-p125) differed at certain crucial positions like position 2 and 9, which are considered as the anchor positions for peptide capturing of the HLA-A*02 allele (Figure 7A).

In functional assays, VLPs (both JCV and BKV derived) were used to prime CD8^+^ T cells in a criss-cross manner. IFN-γ release was measured by ELISPOT after subsequent restimulation with peptides derived from both, JCV or BKV, respectively (Figure 7B). The BKV-p125 peptide was not processed and did not stimulate any T cell response (Figure 7C, 7D). This might be most likely due to the absence of appropriate anchor molecules in the peptide. But both JCV epitopes and BKV-p296 were naturally processed and T cell responses in cross-reactive manner were initiated (Figure 7C, 7D).

## DISCUSSION

In this study, we characterized CD8^+^ T cell immune responses to the VP1-protein derived from JCV which is considered as one of most immunogenic proteins of the JCV [26, 27] as well as of the BKV [28–30] proteome. VP1 represents a major capsid protein and is able to self-assemble into VLPs [31, 32], which are similar to the native virus in morphology [33] and immunology [34, 35]. Some previous studies used virus-infected cell lysates to study virus-specific T cell responses [36, 37]. Such cell lysates are easy to use and allow presenting of all the proteins of virus; however, there are issues of bio-safety and standardization. VLPs, on the other hand, can be handled safely similar to peptides and are used at a lower bio-safety level. By using VLPs in our stimulation assays, we identified several HLA-A*02 T cell epitopes which were naturally processed. Due to the variability in the magnitude of T cell immune responses by responding donors, we selected immunodominant epitopes which elicited immune responses in more than 30% of healthy donors (Figure 3A). By fine mapping of two immunodominant T cell specificities we found the corresponding novel T cell epitopes. They were characterized towards developing future diagnostic and vaccination approaches.

JCV-specific T cell immune responses are believed to be important in controlling viral replication and progression. In particular, CD8^+^ T cells are considered to be the key determinant for survival of PML patients. This is mainly due to their co-localization with JCV-infected brain cells in PML lesions and secondly because of their presence in almost 90% of PML survivors when compared to PML progressors [7, 38–40]. CD4^+^ T cells, on the other hand play crucial role in T cell help by means of cytokines secretion and activating other immune cells. The importance of T cells has been described in the setting of PML, where brain infilterating CD4^+^ and CD8^+^ T-cell sub-populations has been demonstrated to recognize VP1-derived peptides in MS patients [41]. The prevalence of circulating polyomavirus-specific T cells is rare [42] compared to the high seroprevalence (>80%) in adult population. This is probably due to occasional replication of the virus at the sites of latency [43] and due to the absence of viremia [44] thus resulting in low numbers of memory T cells in peripheral blood. Despite their low frequency, T cells seem to be very effective in controlling viral infection in healthy individuals and in allowing recovery of PML patients with HIV infection in whom PML is not as lethal as in patients after allo-HSCT [45–47]. In our study, we employed MLPC assays for efficient induction of peptide-specific responses. This allowed us to detect even low frequencies of JCV-specific T cells in healthy donors which otherwise were not detectable when using fresh, un-sensitized T cells. In this process, we focused only on CD8^+^ T cell responses, but not on CD4^+^ T cell responses. The latter has been investigated by Jelcic et al. wherein the role of JCV-specific CD4^+^ T cells has been described in healthy donors [26]. Another study emphasized on the association of MHC class II variants on JCV infection [48]. Additionally, T2 cells were used to confirm HLA-A*02 restriction during epitope mapping, a strategy which has been used previously for CMV [49]. Overlapping peptides have been used in T cell epitope mapping studies for several viral organisms [50–52]. In our study, the use of a peptide library covering the entire JCV VP1-protein with pentadecamer peptides with an overlap of eleven amino acids ensured that the majority of potential epitopes were included. Moreover, the matrix pool approach allowed us to measure T cell responses with a limited number of immune cells (Figure 2).

Furthermore, we assessed the functional capacity in terms of cytokine secretion by epitope-primed CD8^+^ T cells specific for JCV. We observed a low frequency of JCV/BKV specific cells which were phenotypically characterized as T_EM_ cells (CD45RA^-^, CCR7^-^) and this is in line with previous observations [42]. The virus-specific T cell responses showed mostly the T_EM_ phenotype with a significant release of pro-inflammatory effector cytokines (IFN-γ, TNF-α, and IL-2) upon stimulation with our candidate JCV-specific epitope peptides (Figure 6A, 6B). The production of TNF-α and IL-2 indicate the differentiation of CD8^+^ T cells from an effector to a memory phenotype [53, 54].

Because of the high predominance of latent JCV infection in healthy individuals, there is a high likelihood of (re)activation once the active immune system is suppressed as it is in patients after transplantation or patients suffering from certain diseases like acquired immunodeficiency syndrome (AIDS). Adoptive immunotherapy is one approach that seems to be effective in reconstituting the body with sufficient virus-specific immune cells. This strategy has been fairly successful in treating infections caused by CMV, EBV and adenoviruses in immunocompromised patients. However, only a single case study reported on successful application of JCV-specific T cells to a PML patient [18]. This might be most probably due to late diagnosis of PML in immunosuppressed patients. Knowledge of particular immunodominant T cell epitopes can be very useful to prepare peptide vaccines [1, 27] or designer T cells or a bank of HLA-matched T cell lines that can be readily administered at the time of diagnosis [55–57]. Our group had shown that GMP-compliant preparation of virus-specific T cells can also be achieved using advanced streptamer technology [23]. Recently, other groups reported on a GMP-compliant protocol of cytokine catch analysis for CMV, EBV and adenovirues [21].

We presented two novel immunodominant HLA-A*02-restricted T cell epitopes which, most importantly, are naturally processed. Although, our study is limited to one prominent allele expressed in about 50 % of the Caucasian population, this approach can be utilized to characterize more HLA-dependent epitopes restricted on other HLA types.

While we only investigated samples from healthy blood donors, however, these results provide a good experimental basis for the enrichment of JCV or BKV-specific epitopes across HLA-allele for immunotherapeutic approaches including peptide vaccination and monitoring JCV or BKV-specific T cell responses due to their immunodominance in healthy donors.

Furthermore, with the aim of virus-specific donor leukocyte infusion (DLI), testing of donor lymphocytes towards specificity for particular JCV/BKV epitopes is completely sufficient. Further testing of APCs from patients with a detectable viral load in the APCs will be almost impossible from patient with acute infection with BKV/JCV. Isolation of PBMC from blood of these patients will not result in asservation of such virus infected APCs. This would rather require a screening procedure of the urine bladder or epithelial cells. This was assumed as not to be ethical.

In summary, we defined novel JCV/BKV derived CD8^+^ T cell epitopes to stimulate or select T cells ex vivo for adoptive transfer of virus-specific T cells with the aim of clearance of the viral load. This will broaden the therapeutical option for patients after allogeneic stem cell transplantation suffering from polyomavirus reactivation. A potential strategy for the translation application of our novel epitopes into clinical practice is to combine them with already described epitopes to stimulate DLI ex vivo and to use cytokine-secretion or/and streptamer technologies to produce multi-epitope DLI. This would be an ideal strategy to avoid escape mechanisms and to cover majority of the patient population.

## MATERIALS AND METHODS

### Blood samples from healthy donors

Buffy coats from healthy donor blood samples were obtained from the blood bank of the Institute for Clinical Transfusion Medicine and Cell Therapy (IKTZ, Heidelberg, Germany) after receiving informed consent. Peripheral blood from healthy donors was obtained from Department of Transplantation Immunology. PBMCs were separated from peripheral blood samples by density gradient centrifugation using Ficoll-hypaque solution (Biochrom, Berlin, Germany). PBMCs were cryopreserved in 90% heat-inactivated fetal bovine serum (FBS, Gibco^®^, Grand Island, NY, USA) plus 10% dimethyl sulfoxide (DMSO, Sigma-Aldrich, St. Louis, MO, USA) and stored in liquid nitrogen until use.

### HLA-typing

Typing for HLA-A*02 was performed by staining PBMCs with an anti-HLA-A*02 antibody (Clone BB7.2, Biolegend, San Diego, CA, USA) for 20 min at 4^°^C, in the dark. Flow cytometric acquisition was performed with a FACS LSR II cytometer and data were analyzed using BD FACSDiva software (Becton Dickinson, New York, NY, USA).

### Peptides and proteins

Pentadecamer peptides (OP-1 to OP-86) with an overlap of eleven amino acids covering the entire VP1-protein of JCV as well as nonamer peptides were synthesized and provided by peptides&elephants GmbH (Postdam, Germany). Overlapping pentadecamer peptides were arranged in a horizontal and vertical way to prepare a matrix encompassing 19 peptide matrix pools (MP 1 to 19), thereby, each peptide was present in two different peptide matrix pools.

VP1 VLPs derived from JCV and BKV were produced in yeast and prepared as previously described [24]. BKV and JCV VP1 VLPs were used to confirm the natural processing of antigens.

### Mixed lymphocyte peptide cultures (MLPC)

MLPC were performed using fresh or thawed PBMCs as described earlier with some modifications [58]. Briefly, CD8^+^ T lymphocytes were positively isolated from PBMCs using magnetic cell separation (MACS) CD8 microbeads (Miltenyi Biotec, Bergisch-Gladbach, Germany). CD8^-^ PBMCs were irradiated with 30 Gy and loaded at 37^°^C either with JCV/BKV nonamer/pentadecamer peptides (1 μg/ml), peptide pools (1 μg/ml) or VLPs (10 μg/ml). CD8^+^ and CD8^-^ PBMCs (ratio 1:4) were then co-cultured in culture medium supplemented with 2.5 ng/ml Interleukin-2 (IL-2, Sigma Aldrich, Steinheim, Germany) and 20 ng/ml IL-7 (Miltenyi Biotec) for one week.

### Flow cytometry/Intracellular cytokine staining

Intracellular staining for the cytokines Interferon-γ (IFN-γ), Tumor necrosis factor α (TNF-α) and IL-2 was performed as originally mentioned by Jung *et al*. [59]. Briefly, cells after one week MLPC were restimulated with appropriate antigens in the presence of Brefeldin A for six hours (Biolegend, San Diego, CA, USA). Cells were harvested for live-dead NEAR (Invitrogen, Thermo Fisher Scientific, Waltham, MA USA) and surface marker staining followed by fixation using paraformaldehyde (PFA, Sigma Aldrich). For surface marker staining the following antibodies were used: CD3 Alexa fluor700 (Biolegend, Fell, Germany), CD8 Peridinin chlorophyll (PerCP, Biolegend), CD197 (CCR7) phycoerythrin Cy7 (eBioscience, San Diego, CA, USA) and CD45RA fluorescein isothiocyanate (FITC, BD Biosciences, Heidelberg, Germany). After permeabilization cells were blocked by FcR blocking reagent (Miltenyi Biotec) then stained for intracellular cytokines using anti-IFN-γ PE, anti-TNF-α Pacific Blue and anti-IL-2 AmCyan antibodies (BD Biosciences, Heidelberg, Germany). Samples were measured with the flow cytometer BD LSR II and analyzed using the BD FACSDiva analysis software (BD Biosciences).

### Interferon γ enzyme linked immunospot assays (IFN-γ ELISPOT)

IFN-γ ELISPOT assays were performed as described earlier [58]. Briefly, after pre-wet activation using 35% ethanol ELISPOT plates (Multiscreen IP 96-well plates, Millipore, Massachusetts, USA) were coated with anti–human IFN-γ antibody (Mabtech, Nacka Strand, Sweden). The next day, plates were blocked and loaded with APCs (5 x10^4^ cells/well) and responder cells (5 x10^4^ cells/well). Usually T2 cells (HLA-A*02-positive cells) incubated with the respective peptides served as APCs [60]. T2 cells without any peptides were used as negative control. Stimulation with staphylococcal enterotoxin B (SEB) was used as positive control.

After overnight incubation, washing and incubation with secondary antibody (IFN-γ detection antibody, Mabtech), plates were developed using horseradish peroxidase (HRP) substrate (Becton Dickinson). Visible spots were automatically counted and analyzed using an ELISPOT reader (CTL-Europe GmbH, Bonn, Germany).

Cells were evaluated in triplicates in each ELISPOT assay and mean and standard deviations were calculated.

IFN-γ secretion of T cells was counted in spot forming units (SFU). Positivity in the assay was defined as 2-fold beyond background SFU.

In some experiments antigen-specific responses were characterized by a stimulation index (S.I.) for comparability of different donors in variable experiments. The S.I. represents the ratio of the number of IFN-γ secreting cells after antigen stimulation to that without stimulation (negative control). Responses were considered as positive when the S.I. was equal or above the cut-off of two.

### Cytotoxicity assays

Calcein-acetoxymethyl (AM) cytotoxicity assays were performed as described elsewhere [61]. Briefly, 10^6^/ml T2 cells (target cells) were stained with 10 μg calcein AM (Sigma Aldrich, Steinheim, Germany) for 30 min at 37^°^C with occasional shaking. After three washes, cells were adjusted to 5 × 10^4^/ml and 100 μl were added in V-bottom 96-well plate (Thermo Fisher Scientific, Roskilde, Denmark) as triplicates. Effector cells were harvested from the tissue culture plate, washed and co-cultured with target cells. Target cells with and without triton X-100 (Sigma Aldrich, Steinheim, Germany) were considered as maximum and spontaneous release samples, respectively. After incubation at 37^°^C for 4 h supernatant were transferred into black F-bottom 96-well microplates (Greiner Bio-One, Frickenhausen, Germany) and measured using a Glomax microplate reader (Promega, Madison, WI, USA). Percent specific killing (%) was calculated using the following formula as described earlier [61]: (test release – spontaneous release)/(maximum release – spontaneous release) × 100.
